# Dynamic Behavior and Energy Absorption of Typical Porous Materials under Impacts

**DOI:** 10.3390/ma17205035

**Published:** 2024-10-15

**Authors:** Kui Xie, Menglong Li, Jianghua Shen

**Affiliations:** 1 The 38th Research Institute of China Electronics Technology Group Corporation, Hefei 230000, China; 2022120196@mail.nwpu.edu.cn; 2 School of Aviation, Northwestern Polytechnical University, Xi’an 710072, China; 3 School of Aeronautics, Northwestern Polytechnical University, Xi’an 710072, China; menglongli@mail.nwpu.edu.cn

**Keywords:** porous structural material, impact behavior, relative density, discontinuous medium, finite element method

## Abstract

Porous materials are known for their excellent energy absorption capability and, thus, are widely used in anti-impact applications. However, how the pore shape and size impact the failure mechanism and overall behavior of the porous materials under impact loading is still unclear or limitedly touched. Instead of using homogeneous solids for the porous material model, pores with various shapes and sizes were implanted in a solid to establish the porous materials that have true porous structures, which permits exploration of the local failure mechanism. The results revealed that differently shaped holes have two different dominant deformation modes. And due to their different local stress distributions, they enter the plastic phase earlier and, thus, have higher specific energy absorption. Meanwhile, the model changes from hardening to a quasi-zero stiffness model as the hole size increases. The application of this work can be extended into the field of impact resistance.

## 1. Introduction

Porous structural materials are a class of materials with many pores inside. These pores give these materials special physical and chemical properties [1,2,3,4], such as large specific surface area, high porosity, good absorption capacity, and low density. Due to their large specific surface area, porous structural materials are widely used in gas absorption and liquid separation and as catalyst carriers. Girolamo Costanza [5] has given a detailed description of the properties and applications of porous materials. In the field of energy absorption, more than 60% of the material structures in this market consist of porous materials, and two main types of porous materials exist: flexible with open cells and stiff with closed cells. At the same time, porous materials tend to have flatter stress amplitudes when subjected to impacts, also making them popular in the field of energy absorption [6].

As energy absorption components, the capacity and behavior of those structures under impact loading conditions are practically important. Therefore, it is necessary to understand the mechanical properties of different types of porous structural structures under impact loading [7,8,9,10]. For example, Meng and his coauthors [11] studied the dynamic properties of aluminum-filled foam tubes and obtained their failure mode, impact force history, displacement history, and permanent deformation. F. Saleem et al. investigated the effect of strain rate (SR) and relative density (RD) on the mechanical behavior of metallic foams [12]. A few studies have revealed that the destruction of pores under dynamic loading involves the bending and flexing of cell walls [13,14,15]. Researchers have also investigated the impact properties of lattice structures or matrices of porous structures [16,17,18,19]. For example, Yue et al. proposed a series of new bi-directional gradient lattice structures (BD-GLSs) to improve their tilt impact resistance, which can be used to satisfy the requirement of the crashworthiness of lightweight structures under different impact directions [20]. Besides, in order to maximize the energy absorption properties for lightweight design, K. Kappe et al. optimized the design variables such as relative density and density gradient of the lattice structure by considering different topologies [21]. Meanwhile, C.J. Ejeh et al. [13] investigated the anisotropy and lattice randomness for a triple periodic minimal surface (TPMS) under impact loading. The use of mathematical methods such as simulations is widely used in research on impacts. Liu [22] and Chen Long [23] et al. investigated the mechanical mechanisms of foam materials under impact by numerical calculations. It is no coincidence that more and more researchers are using simulation for their studies [24,25,26,27], which shows the reliability of those calculations.

Most of the current research about porous materials with anti-impact properties focuses on the structural design at various scales. In those studies, the porous materials are described as a homogeneous material, and only the relative volume fraction is used to define the porous material. As in the derivation of the fluctuation equations, the material is a continuous homogeneous medium, which leads to the following equation:

(1) $$\frac{\partial^{2}u}{\partial t^{2}}=c^{2}\nabla^{2}u$$

However, due to the unique characteristics of porous materials with high porosity, the cross-sections are often found to be discontinuous, and Equation (1) is no longer applicable. In order to deal with this characteristic, Yuan et al. [28] proposed stress wave governing equations in the mesoscopic discontinuous medium.

(2) Dx+dxαixCEi*Dx+dxαixCux,t=ρ0i*∂2ux,t∂t2

(3) Dx+dxαixC1ρ0i*Dx+dxαixCσx,t=1Ei*∂2σx,t∂t2

where DxαaCfx=1Γ1−α∫axf′(τ)x−ταdτ is the fraction derivative defined by Caputo [29]. α is the fractional order, $\rho_{0}^{*}$ is the equivalent fractional density, and $E^{*}$ is the equivalent fractional modulus. Equivalent density and equivalent Young’s modulus are also used in Equations (2) and (3) to approximate porous materials.

Instead of direct equivalence calculations for porous materials, some researchers have begun to analyze the performance of structures with respect to the shape and distribution of openings, and M. Rezasefat [30] has analyzed the effect of the diameter of the openings and the number of openings in a laminate subjected to a low-velocity impact. Huayan Chen [31] analyzed the performance of high-strength steel beams with web openings under impact. Numerous studies have demonstrated the value of mechanically analyzing structures for the shape and size of openings to facilitate their application and investigate service value in many different environments. At present, the impact dynamics analysis of the geometrical configuration and topology of the holes is mainly carried out in beams and plates [32,33,34,35,36], and the impact dynamics analysis of porous structural materials, as mentioned earlier, is simply measured by the relative density, with less analysis of the specific effects of the holes.

Therefore, to investigate the influence of the shape, size, and distribution of holes on the impact response of porous materials, this work investigates their dynamic problems by establishing the array spherical hole model and the square hole model. Based on the existing experimental data, combined with finite element analysis, we analyze its mechanical response and deformation behavior under dynamic loading. And the following characteristics were studied:

(1) Investigating the influence of the shape and size of the openings on the deformation form and mechanical response of porous materials;
(2) Explaining the connection between the deformation forms and energy absorption capacity of different porous materials;
(3) Exploring the application value of the characteristics of porous materials (quasi-zero rigidity phenomenon in this study) in the field of energy absorption.

It aims to provide a new method for the optimization of porous structural materials and related mechanical behavior studies.

## 2. Materials and Methods

This section details the design and simulation verification process of variable cross-section and porous materials based on Ti-6Al-4V materials.

### 2.1. The Parameters of the Ti-6Al-4V Specimen

At higher strain rates, there is a significant increase in the dynamic stress of the material compared to the static state, a phenomenon found in many metals. At the same time, metallic materials exhibit a significant thermal softening effect at high temperatures. Based on the above factors, the Johnson–Cook equation was proposed.

(4) $$\sigma =\left(A+B\varepsilon_{e}^{n}\right)\left(1+C\ln{\dot{\varepsilon }}_{e}^{*}\right)\left(1-T^{*m}\right)$$

(5) $$\varepsilon_{f}=\left[D_{1}+D_{2}\exp\left(D_{3}\sigma^{*}\right)\right]\left[1+D_{4}\ln{\dot{\varepsilon }}_{e}^{*}\right]\left[1+D_{5}T^{*}\right]$$

where A, B, n,C,m,D1,D2,D4,D4,D5 are the material properties; σ is the Mises flow stress; $\varepsilon_{e}$ is the equivalent plastic strain; ${\dot{\varepsilon }}_{e}^{*}$ is the strain rate; ${\dot{\varepsilon }}_{0}$ is the quasi-static strain rate; ${\dot{\varepsilon }}_{0}=1.0\;s^{-1}$; $T^{*}=(T-T_{room})/(T_{melt}-T_{room})$; T is the experimental temperature; $T_{r}$ is the room temperature; and $T_{m}$ is the melting temperature.

In this paper, the computational analysis was carried out using Ti-6Al-4V, and by fitting the experimentally obtained data, the Johnson–Cook model parameters were obtained as shown in Table 1 [37].

Meanwhile, the remaining mechanical, physical property parameters of the material are shown in Table 2 [37].

**Figure 1:**
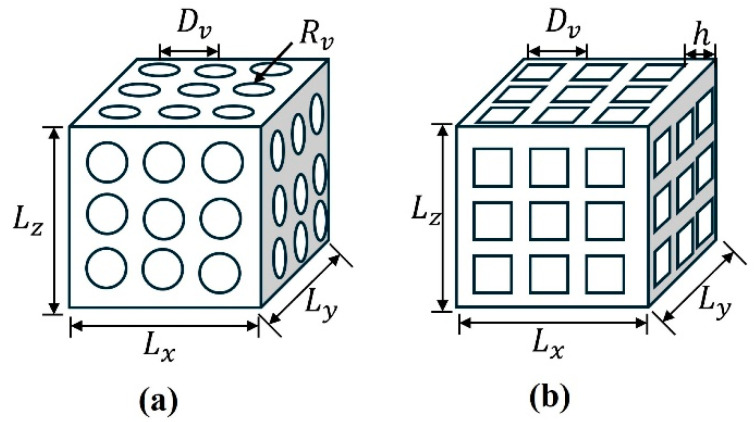
The test specimens of 2 different types. (**a**) Spherical hole model and (**b**) square hole model.

Based on the above matrix materials, this paper for different constant cross-section porous structures and variable cross-section porous structures is defined as shown in Figure 1. Figure 1a shows the reference specimen with the size of $L_{x}=3\;mm,\;\;L_{y}=3\;mm,\;\;L_{z}=2.5\;mm$, which is a rectangular porous specimen with arrays of spherical holes where the distance between the holes is $D_{v}$ and the radius of the holes is $R_{v}$. And Figure 1b shows the square hole model where the distance of holes is $D_{v}$, and the length of the square is h. The specimens and dimensions analyzed are shown in Table 3.

### 2.2. Simulation Calculation Method

**Figure 2:**
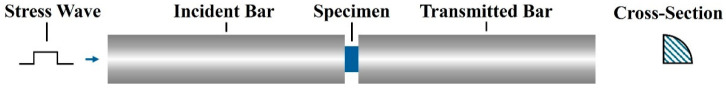
Structure diagram of Hopkinson pressure bar.

For dynamic mechanical analysis, the Hopkinson bar is usually used for experiments, and this method is one of the most important techniques for the study of dynamic properties of materials nowadays. Therefore, in this paper, based on the above experimental method, ABAQUS/explicit 6.14 is used to simulate and analyze the dynamic impact of the structure described in Section 2.1, and the simulation model assembly schematic is shown in Figure 2. In order to reduce the computational accuracy, a quarter model is used, and the model interface is defined as a symmetric cross-section. A Ti-6Al-4V bar with a diameter of 13 mm is selected for the incident and transmitted bar. According to the one-dimensional stress wave theory, in order to ensure that the used specimen is always in stress equilibrium during the testing process, the loading time should be much longer than the time required for the stress wave to travel back and forth within the specimen once. The length of the loading direction of the specimen is 2.5 mm, and the wave speed of the specimen is $C_{s}=\sqrt{\frac{E}{\rho }}=4944\;m/s$, so the loading time should be much longer than 1 μs, and the loading time of 0.1 ms is used in this calculation.

**Figure 3:**
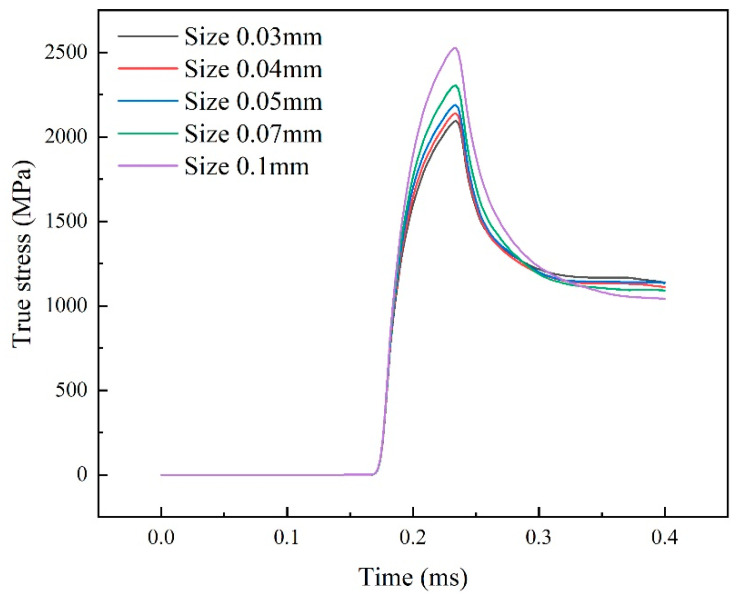
Mesh size and average stress on the transmissive end face.

During the numerical simulation of impact processes, cells often fail due to deformation as a result of large loads. In the analysis using ABAQUS/explicit, the above Johnson–Cook failure model is used for mesh deletion. However, a mesh with too much distortion still occurs, which leads to errors in the calculation. Therefore, it is necessary to use smaller cells for meshing to improve the precision and accuracy of the calculation, but the actual calculation process is limited by the calculation time. It is impossible to divide the test piece into dense mesh, so it is necessary to verify the mesh irrelevance. Mesh sizes of 0.1 mm, 0.07 mm, 0.05 mm, 0.04 mm, and 0.03 mm, respectively, were dynamically loaded at a strain rate of $5000\;s^{-1}$. The average Mises stress at the transmissive end face of the specimen was used as a verification method, and the strain time curves obtained from the calculations for different mesh sizes are shown in Figure 3.

As can be seen in Figure 3, the average Mises stresses on the transmissive end faces of the specimens tend to be the same as the mesh density is gradually reduced from 0.1 mm, and the resultant images almost converge at mesh sizes of 0.03 mm and 0.04 mm. Comparing the maximum values of the loading history in the graphs, the structure is obtained as shown in Table 4.

The mesh size of 0.03 mm is used as the reference size, and the relative error between the mesh of 0.04 mm and 0.05 mm is less than 5%. Therefore, a mesh size of 0.03 mm is preferred in this finite element calculation. Meanwhile, the geometry of the computational example is relatively simple, and hexahedral mesh (C3D8R) is used in order to ensure accuracy and speed up the computation.

## 3. Results and Discussion

### 3.1. Dynamic Response of Array Spherical Hole Models

**Figure 4:**
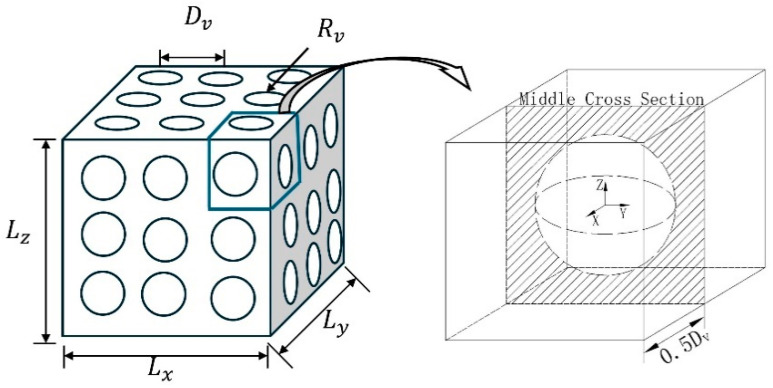
The cross-section of an array of spherical models.

The model cross-section with arrayed spherical holes is shown in Figure 4, which has a variable cross-section. The model is partitioned into repetitive cells based on the array distance $D_{v}$. The cross-sectional area and cell volume of its cells are now defined and calculated as follows.

(6) $$A(R_{s})=\left\{\begin{array}{c} D_{v}^{2}-\pi R_{s}^{2}\;2R_{s}\leq D_{v}\\ D_{v}^{2}-\pi R_{s}^{2}+2R_{s}^{2}\mathrm{acos}\frac{D_{v}}{2R}-R_{s}\sqrt{4R_{s}^{2}-D^{2}}\;\sqrt{2}R_{s}\leq D<2R_{s}\\ 0\;D<\sqrt{2}R_{S} \end{array}\right.$$

where $A_{s}$ is the cross-sectional area and $R_{s}$ is the radius of the circle corresponding to the cross-section. According to Equation (6), the volume equation of the array spherical hole model can be obtained as follows:

(7) $$V=\int_{-0.5D_{v}}^{0.5D_{v}}A\left(\sqrt{R_{v}^{2}-x^{2}}\right)dx$$

**Figure 5:**
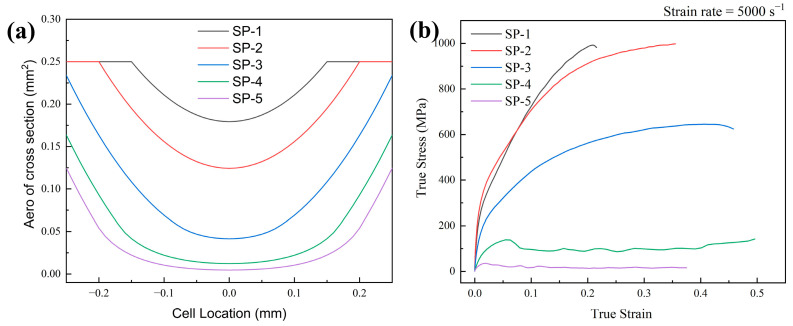
(**a**) The aero of cell cross-section; and (**b**) The dynamic response of array of spherical models from SP-1 to SP-5.

The cross-sectional area of the cell calculated by Equation (6) is shown in Figure 5, and the cross-sectional area decreases with the increase in the radius of the spherical aperture, and the whole cell is converted from a closed cell to an open cell when D<2R. According to Equation (6), the limit value of its open-hole radius is $\sqrt{2}R_{s}=D$.

**Figure 6:**
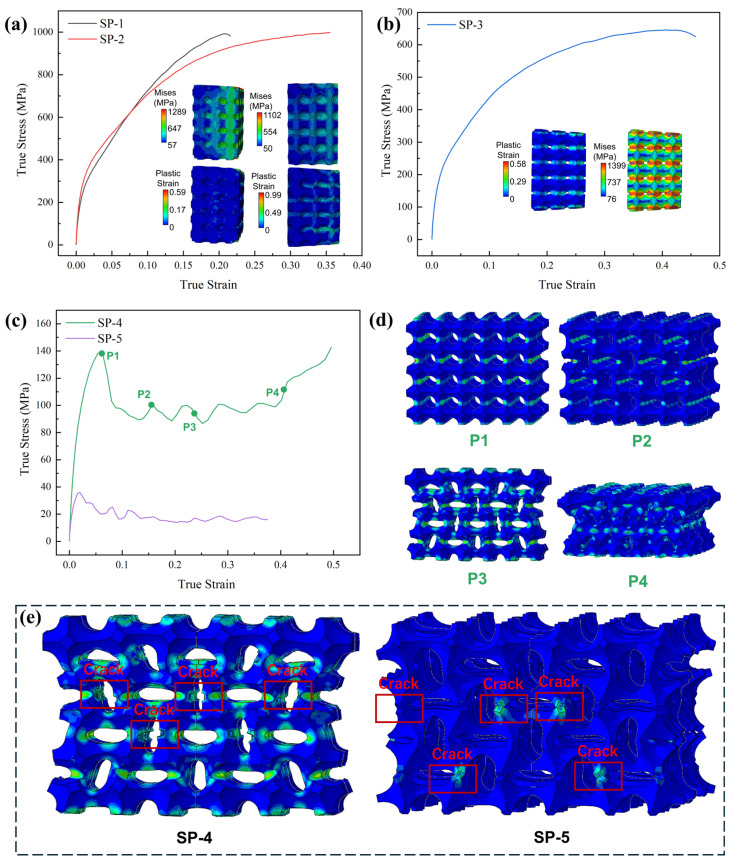
(**a**–**c**) The simulation results of the array spherical models from SP-1 to SP-5; (**d**) the deformation model of SP-4; and (**e**) the crack of SP-4 and SP-5.

Figure 5b and Figure 6 show the stress–strain curves and cloud diagrams of the array hole models under impact with a strain rate of $5000\;s^{-1}$. Model SP-1 and model SP-2 correspond to closed cells, and model SP-3 to model SP-5 correspond to open-cell models. The stress–strain curves corresponding to SP-1 and SP-2 do not differ much, and the main deformation form of SP-1 is dominated by vertical compression through the stress–strain comparison in Figure 6a. In SP-2, the stresses are mainly distributed in the middle of each cell (i.e., at the smallest cross-sectional area in Figure 5a), i.e., the diameter part of the spherical hole. It can be predicted that the trend will be more pronounced as the radius of the spherical aperture increases. Figure 6b shows the stress–strain curve of the open-cell unit, whose stress concentration location is closer to the open-hole section, while the structure still exhibits compression-dominated deformation. It can be clearly seen in Figure 6c that the dynamic response form is different from the previous three structures in that after the structure reaches the yield point for the first time, the stress decreases rapidly, and a softening phenomenon occurs. However, a certain degree of rebound of the stress occurred again in the follow-up. Based on the stress–strain curves, the points (P1–P4) taken for the SP-4 structure were observed. In the vicinity of its first yield point, the plastic strain cloud at point P1 (Figure 6d) shows an approximately uniform deformation, concentrated in the center of the cell, but two larger strains appear at the second layer of the cell. With further sub-propagation of the stress wave, the specimen produces bending deformation along the larger strains, which deforms and twists the circular unit hole into an ellipse (P2), at which point, the specimen enters a relatively stable state again and the stress rises back. With further increases in strain, each layer of cells produced large torsional deformation, and eventually, the whole experiment was compressed into a dense structure. Therefore, in the last part of the loading, the stresses applied to the structure are further elevated, forming a rising section after the P4 point. Like the SP-4 structure, the SP-5 structure also has such a phenomenon, which in turn, produces the upward and downward fluctuation of the stress–strain curve. Since the fluctuation is small, when used as an energy-absorbing structural member, the outer part will remain in a relatively stable stress state during the loading of this part. This phenomenon proves to be beneficial as a stable energy-absorbing structure, i.e., quasi-static stiffness structure.

**Figure 7:**
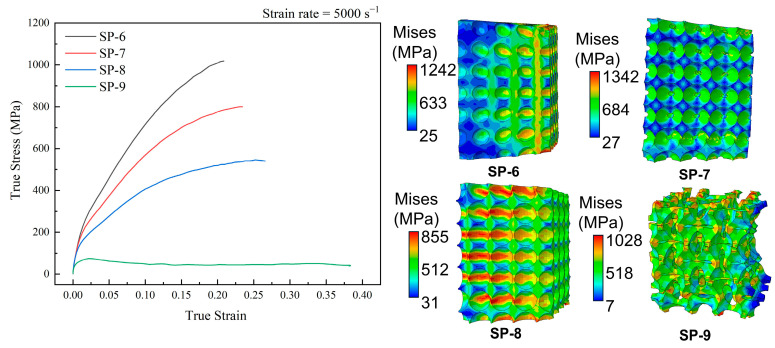
Stress–strain curves and stress cloud diagrams.

Meanwhile, in the SP-4 and SP-5 models, a significant crack was generated on the tensile side due to the excessive torsional deformation between the cells, as shown in Figure 6e. The appearance of this crack is the main reason for the stress decrease in SP-4 and SP-5. And as the size of the pores increases, the structure produces a larger fracture area and more cracks. This phenomenon is also present in Figure 7. When the structure is a closed cell, according to the stress cloud diagram, the overall structure shows compressive deformation dominance, the outer side of the model is subjected to a larger stress and shows contraction to the inner side in the form of a 45° angle, and the SP-6 model in Figure 7 is similar to the SP-1 and SP-2 models in Figure 6. This phenomenon indicates that the edges are subjected to a larger stress distribution when the structure is subjected to impact loading.

When the hole inside the model becomes larger and reaches $2R_{S}>D$, i.e., an open cell, the stress is gradually concentrated at the center of the open hole (SP-7). And with the gradual increase in the open hole and the cracking at the stress concentration (SP-8 and SP-9), the deformation form is gradually transformed into bending-deformation-dominated.

From the above, it can be seen that the deformation model changes from compression-dominated deformation to bending-dominated deformation when the structures change from a closed cell to an open cell. Moreover, the structure exhibits a softening phenomenon and tends to a quasi-zero stiffness deformation mode.

### 3.2. Dynamic Response of Square Hole Model

**Figure 8:**
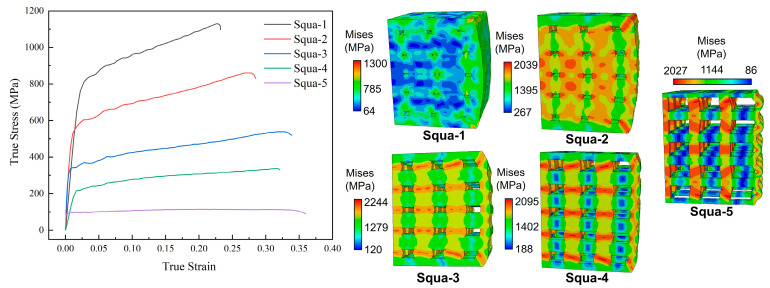
Stress–strain curves and cloud diagrams of rectangular hole structures.

As analyzed in Section 3.1, for the variable section hole model subjected to impact loading, the stresses are concentrated in the part with the smallest cross-sectional area. The size of the cross-sectional area will affect the deformation form of the structure and cause the structure to fail. If the structural holes are set as regular squares, the calculated stress–strain curves and cloud diagrams are shown in Figure 8. Unlike the array spherical models, the deformation form of the rectangular hole model is always compression-dominated. It can be equally observed via Squa-1 and Squa-2 in Figure 8 that the profit is distributed mainly at the edges of the structure. As the hole in the structure increases, the layered bending at the edges becomes apparent, eventually resulting in a bending deformation as shown in Squa-5. Unlike the array spherical model, the overall structure does not show instability and fracture. However, quasi-zero stiffness deformation also occurs.

### 3.3. Energy Absorption Capacity with Relative Density

For energy absorption performance, specific energy absorption (SEA) is usually used. SEA is defined as follows [38]:

(8) $$SEA=\frac{\int_{0}^{\delta }Fd\delta }{m}$$

**Figure 9:**
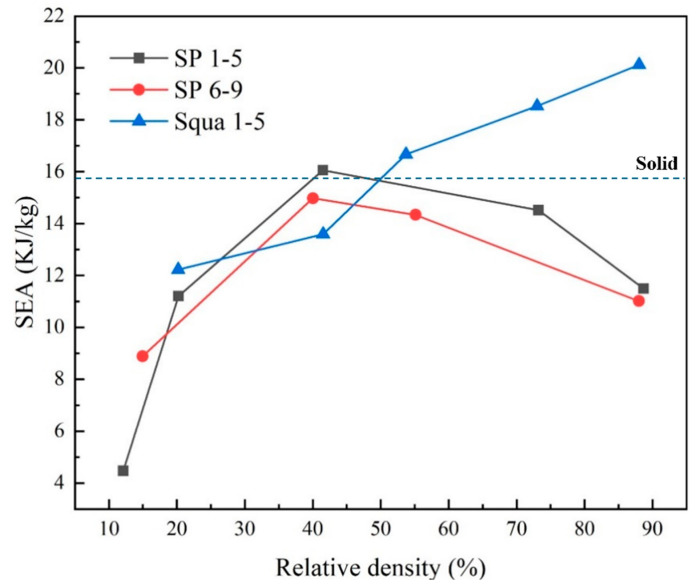
Comparison of relative density with SEA for different structures.

By calculating the SEA of the above different structures at different strains, Figure 9 is obtained. At the same time, the deformation is not only related to the macroscopic structure but also to the microstructure of the materials. In this study, the material is homogeneous (without considering the microstructure). Moreover, in order to exclude the influence of the Ti-6Al-4V materials on their energy absorption, the dynamic mechanical behavior of the solid material was calculated, and its SEA value was obtained, as shown in Figure 9.

In the spherical hole models, the SEA value first rises and then decreases with the increase in porosity, reaching an extreme value at a relative density of 40%. Through Section 3.1, at $2R_{s}<D_{v}$, the structure shows compression-dominated deformation; at $D_{v}<2R_{s}$, the structure tends to bend-dominated deformation. And its SEA reached its maximum value when shifting from a closed-cell to an open-cell structure.

The SEA value is approximately proportional to the porosity in square hole structures, both open-cell and closed-cell. The main reason for this is that the deformation is always compression-dominated under impact. However, when the spherical hole structure is transformed into a bending–absorbing dominant deformation, the plastic strain that occurs earlier due to the stress concentration becomes the main form of energy absorption, which in turn makes the SEA value of the hole structure larger than that of the rectangular structure.

To determine the transform point of a porous material, a stress concentration factor is used. Meanwhile, according to Equation (9), the stress concentration factor ($K_{s}$) of two typical models and a solid model (as shown in Table 5) is calculated as follows:

(9) $$K_{s}=\frac{\sigma_{\max}}{\sigma_{avg}}$$

From Table 5, it can be found that the spherical array structure has the largest $k_{s}$, so plastic deformation is easier and, therefore, it is more likely to have a higher SEA. However, by observing the stress–strain curve of the spherical array structure and the square structure, it can be seen that the square has a higher Young’s modulus. So, in the case of small deformation, the square hole models with the same strain have a greater SEA value. Therefore, in Figure 9, the SEA value is greater than that of spherical hole models.

Meanwhile, although the solid structure has the smallest stress concentration factor, its SEA value is not large. Comparing the solid and porous materials, it can be shown that the structural form of the porous material plays an important role in its energy absorption.

Moreover, we compare and discuss the work of this paper with the work of the rest of the researchers. Most of them study the porous properties of lightweight materials such as aluminum alloys, foams, and TPUs. And less work has been performed on the porous calculations for Ti-6Al-4V, and based on the results in the literature, we make the comparison in Table 6.

## 4. Conclusions

By analyzing porous structures with different pore shapes and sizes, their deformation behaviors under impact and local failure modes were obtained, and the relationship between their mechanical properties and specific energy absorption with the pores was exposed. The following conclusions are obtained.

(1) Porous materials have two main forms of deformation: compression-dominated deformation and bending-dominated deformation. In the spherical hole structure, the deformation form changes from compression-dominated deformation to bending-dominated deformation as the structure transforms from closed-cell ($2R_{s}<D_{v}$) to open-cell ($2R_{s}>D_{v}$). Unlike spherical holes, the deformation in square holes is always compression-dominated, either open-cell or closed-cell.

(2) The shape and size of the holes allow the quasi-zero stiffness phenomenon to occur. As the hole size increases, the porous material transforms into an open-cell model, allowing the strain-hardening phenomenon to disappear rapidly. At low relative densities, the structure receives continuous strain loading while the stress is maintained at a steady value, and quasi-zero stiffness occurs. This phenomenon is very favorable for the application of porous materials in the field of energy absorption.

(3) The specific energy absorption capacity is correlated with the hole shape, size, and deformation mode. For the same relative density and deformation form, square holes have large SEA values due to their smaller stress concentration factor. At small relative densities, circular holes cause the structure to rapidly enter the plastic section due to stress concentration and bending-dominated deformation. The plastic energy absorption capacity is greater than that of the square pore structure, resulting in a higher SEA. For the same form of deformation, a decrease in the relative density implies a decrease in the SEA value.

(4) However, the configurations of porous materials are often random and complex. Due to the low arithmetic power used in this work, the study of energy absorption in porous materials based on random hole distributions will be followed up. Moreover, based on the consideration of practical applications, the phenomenon will be subsequently comprehensively elaborated in multiple aspects through impact experiments and microscopic characterization.

## Figures and Tables

**Table 1:** The Johnson–Cook parameters of the TI-6AL-4V.

A (MPa)	B (MPa)	n	C	m	D1	D2	D3	D4	D5
1077	845	0.58	0.025	0.7538	0.0395	1.0072	1.9234	0.014	3.87

**Table 2:** The parameters of the TI-6AL-4V.

Density $\left(\mathrm{kg}/m^{3}\right)$	Elastic Modulus (GPa)	Poisson’s Ratio
4500	110	0.3

**Table 3:** The parameters of the specimens.

Case	$L_{x},L_{y},L_{z}$ (mm)	$D_{v}$ (mm)	$R_{v}(h)$ (mm)	Volume $(mm^{3})$	Relative Density (%)
SP-1	3, 3, 2.5	0.5	0.150	20.0	88.70
SP-2	3, 3, 2.5	0.5	0.200	16.5	73.21
SP-3	3, 3, 2.5	0.5	0.260	9.34	41.51
SP-4	3, 3, 2.5	0.5	0.300	4.55	20.22
SP-5	3, 3, 2.5	0.5	0.320	2.72	12.11
SP-6	3, 3, 2.5	0.4	0.150	19.8	88.00
SP-7	3, 3, 2.5	0.4	0.190	12.4	55.14
SP-8	3, 3, 2.5	0.4	0.210	9.01	40.03
SP-9	3, 3, 2.5	0.4	0.250	3.36	14.95
Squa-1	3, 3, 2.5	0.5	0.196	19.8	88.02
Squa-2	3, 3, 2.5	0.5	0.165	16.4	73.02
Squa-3	3, 3, 2.5	0.5	0.131	12.1	53.72
Squa-4	3, 3, 2.5	0.5	0.111	9.34	41.52
Squa-5	3, 3, 2.5	0.5	0.072	4.55	20.20

**Table 4:** The relative error between mesh size and maximum stress.

Mesh Size (mm)	Maximum Stress (MPa)	Relative Error (%)
0.03	2095.08	0
0.04	2139.73	2.13
0.05	2189.29	4.50
0.07	1304.19	9.98
0.10	2526.74	20.6

**Table 5:** Stress concentration factors for four structures.

Case	Solid	SP	Squa
$K_{s}$	6.14	13.79	9.56

**Table 6:** A comparison of the work in this paper with other work [20,21,39,40].

Case	Material	SEA (kJ/kg)	Comparison
This Work	Ti-6Al-4V	19.8	
Hangyu Lv 2023	CFRP	0.72	low-velocity impact
Liang Wan 2024	316L	17.8	quasi-static lateral crushing
Zhen Wang 2024	aluminum foam	0.43	quasi-static
Lei Yue 2024	Nylon	5.3	low-velocity axial impact
Konstantin Kappe 2024	Al-Sc-Mg alloy	9.4	Multi-objective optimization; low-velocity axial impact

## Data Availability

The raw data supporting the conclusions of this article will be made available by the authors on request.
